# Conditional knockdown of ectodermal‐neural cortex 1 in excitatory neurons impairs autophagy‐lysosome pathway and accelerates tau propagation *in vivo*

**DOI:** 10.1002/alz70855_102099

**Published:** 2025-12-23

**Authors:** Hongjun Fu, Tae Yeon Kim, Diana M Acosta, Nicholas Sweeney, Sheeny Vo, Min Wu, Bradley T. Hyman, Ralph A. Nixon

**Affiliations:** ^1^ The Ohio State University, Columbus, OH, USA; ^2^ Ohio State University, Columbus, OH, USA; ^3^ Massachusetts Alzheimer's Disease Research Center, Charlestown, MA, USA; ^4^ New York University Langone Health, New York, NY, USA

## Abstract

**Background:**

Higher ectodermal‐neural cortex 1 (ENC1) RNA level is correlated with higher residual cognition in participants of the Religious Orders Study and the Rush Memory and Aging Project. Transcriptomic analyses show decreased overall ENC1 mRNA in human brains with Alzheimer's disease (AD) compared to control brains. We have previously identified ENC1 as one of the hub genes controlling tau homeostasis via the weighted gene co‐expression network analysis of healthy donors. However, the relationship between ENC1 and tau remains unclear. ***We hypothesize that ENC1 interacts with tau and enhances tau clearance via the autophagy‐lysosome pathway (ALP), inhibiting tau propagation***.

**Methods:**

The co‐immunoprecipitation and Duolink assay were used to measure the interaction between human ENC1 and tau *in vitro* and in human brains. The specific knockdown of ENC1 (mainly in the nucleus) in excitatory neurons (ENs) at the superficial layers of adult mouse entorhinal cortex (EC) was achieved by stereotaxic injection of AAV8‐CaMKIIa‐Cre in the EC of *ENC1^flox/flox^* mice. An autophagy reporter mouse (TRGL6) (mRFP‐eGFP‐LC3) and TRGL6;PS19 tau mice were used to measure the effect of ENC1 on autophagy dynamics. The tau propagation was measured by stereotaxic injection of a tau‐propagation reporter virus (AAV8‐hSyn‐RFP‐2A‐V5‐Tau) or TBS‐soluble human tau seeds in the EC of *ENC1^flox/flox^* mice.

**Results:**

We found that ENC1 interacted with both total tau protein (TauC+) and pathological tau (TauY9+), and this interaction was significantly increased in human AD brains compared to controls. Conditional knockdown of ENC1 in ENs of TRGL6;ENC1*^flox/flox^* and TRGL6;PS19;ENC1*^flox/flox^* mice significantly reduced the number of single red puncta (autolysosomes) and increased the yellow puncta (autophagosome and poorly acidified autolysosomes), suggesting the ALP was impaired. Also, the knockdown of ENC1 specifically in ENs of the EC significantly increased the number of V5 only positive tau recipient neurons or MC1‐positive (conformation‐dependent tau) in the spreading areas such as deep layers of the EC and the hippocampal CA1. These results suggest that conditional knockdown of ENC1 in ENs could promote tau propagation *in vivo*.

**Conclusions:**

We demonstrate that ENC1 is a novel modulator of tau protein homeostasis by regulating tau clearance and propagation probably via the regulation of ALP.

